# Prognostic value of mitral annular plane systolic excursion for adverse cardiovascular events in patients with acute myocarditis

**DOI:** 10.3389/fcvm.2026.1733925

**Published:** 2026-03-27

**Authors:** Ruichen Ren, Qingyuan Zhao, Wenting Li, Ying Zhang, Xinghua Xu, Lei Lv, Yang Zhang

**Affiliations:** 1 Department of Radiology, Qilu Hospital of Shandong University, Jinan, China; 2 Department of Radiology, Beijing YouAn Hospital, Capital Medical University, Beijing, China; 3 Department of Ultrasound, Xiajin County People’s Hospital, Dezhou, China

**Keywords:** cardiac magnetic resonance imaging, feature tracking, mitral annular plane systolic excursion, myocarditis, prognostic

## Abstract

**Purpose:**

Mitral annular plane systolic excursion (MAPSE) has prognostic value as a surrogate indicator of ventricular function in cardiovascular disease, but its prognostic value in patients with acute myocarditis is unclear.

**Methods:**

Our cohort included 46 patients with acute myocarditis and 26 healthy controls, all of whom underwent cardiac magnetic resonance. Left atrial and left ventricular strain and MAPSE were assessed using feature tracking, and patients were followed up in a group that experienced a major adverse cardiovascular event (MACE) (*n* = 11) and a group that did not experience a MACE (*n* = 35). Cox regression modelling was used to assess the prognostic value of MAPSE in acute myocarditis.

**Results:**

Left ventricular strain parameters (including global longitudinal, circumferential, and radial strain), left atrial strain parameters (reservoir and conduit strain), and MAPSE were significantly reduced compared with patients without MACE. Receiver operating characteristic (ROC) curve showed that MAPSE had a higher area under the curve (AUC) in identifying MACE. By Kaplan–Meier analysis, the risk of death increased significantly with decreasing lateral and septal MAPSE (log-rank *P* = 0.0025, *P* = 0.0065). After adjusting for clinical and imaging risk factors, age (HR 1.139, 95%CI 1.056–1.228), lateral MAPSE (HR 0.594, 95%CI 0.355–0.955), and septal MAPSE (HR 0.647, 95%CI 0.420–0.995) were significantly associated with MACE. The ROC curves showed that the model including both lateral and septal MAPSE did not improve predictive performance compared to lateral MAPSE alone (AUC = 0.8831 vs. AUC = 0.9095).

**Conclusion:**

MAPSE has prognostic value for adverse cardiovascular events in patients with acute myocarditis, and lateral MAPSE has better predictive performance.

## 1 Introduction

Myocarditis is an acute or chronic inflammatory disease of the myocardium that can be caused by infection, immune response, or toxic injury (1). Symptoms of myocarditis vary from subclinical disease to chest pain with acute coronary syndrome-like manifestations, refractory cardiogenic shock, or sudden cardiac death due to ventricular fibrillation (2, 3). Myocarditis is the leading cause of sudden cardiac death and dilated cardiomyopathy (4, 5).

Cardiac magnetic resonance imaging has become the primary non-invasive method for diagnosing myocarditis and risk stratification (6). Conventional left ventricular ejection fraction (LVEF) is not sensitive enough to assess myocardial systolic function, and it is difficult to identify subtle but important changes in left ventricular (LV) systolic function (7). Cardiac magnetic resonance (CMR) feature tracking can be used to quantify biventricular and atrial deformations, displacements, torsions, and dyssynchrony (8). Several studies have shown that LV and left atrial (LA) strain is impaired in patients with acute myocarditis and can be a predictor of adverse prognosis (9, 10).

Atrial plane motion is an easy-to-measure surrogate for ventricular function, and reduced valve plane motion on echocardiography has been shown to be a predictor of adverse events in patients with a variety of cardiovascular diseases (11, 12). Impaired MAPSE on CMR is an independent determinant of all-cause mortality in patients with reduced LVEF (<50%) and predicts major adverse cardiovascular events in mixed populations, including patients with coronary artery disease or previous myocardial infarction, for major adverse cardiovascular events (13, 14). However, impaired MAPSE in patients with acute myocarditis has not been evaluated to date for its impairment and prognostic significance. Therefore, this study aimed to investigate the correlation of CMR-derived MAPSE with indices of atrial ventricular strain and to assess its predictive value for adverse cardiovascular events in patients with acute myocarditis.

## 2 Methods

This retrospective study was approved by our institutional ethics committee and written informed consent was waived; patient details were not disclosed.

### 2.1 Study population

We retrospectively analysed the medical records of 59 patients diagnosed with clinically suspected myocarditis and who underwent CMR from January 2016 to July 2024. The clinical diagnosis of suspected myocarditis was in accordance with the 2013 European Society of Cardiology guidelines (5). Diagnostic criteria included elevated troponin, ECG abnormalities, and structural or functional abnormalities confirmed by echocardiography or CMR. Patients with cardiac symptoms (chest pain, dyspnea or palpitations) who fulfilled one or more of the diagnostic criteria, as well as patients who did not have any symptoms but fulfilled two or more of the above criteria, were identified as clinically suspected of having myocarditis. Exclusion criteria included coronary artery disease, non-ischemic cardiomyopathy, heart valve disease, Kawasaki disease and congenital heart disease. A total of 48 patients were included.

Baseline characteristics of patients at the time of CMR examination were collected, including demographic characteristics (age, sex, and body mass index [BMI]), cardiovascular risk factors (hypertension, diabetes mellitus), and laboratory findings (N-terminal pro-B-type natriuretic peptide (NT-proBNP), high-sensitivity cardiac troponin I [hs-TNI], white blood cell counts, and creatine kinase (CK)).

All patients were followed up by a radiologist (R.RC., with 2 years of CMR experience) by chart review or telephone interview. The primary endpoint was major adverse cardiovascular events, defined as cardiac death, cardiogenic shock, new-onset heart failure, and readmission for recurrence of myocarditis or exacerbation of heart failure. Two patients (4.07%) were lost to follow-up and were not included in further outcome analyses. 46 patients were finally included.

**Figure 1:**
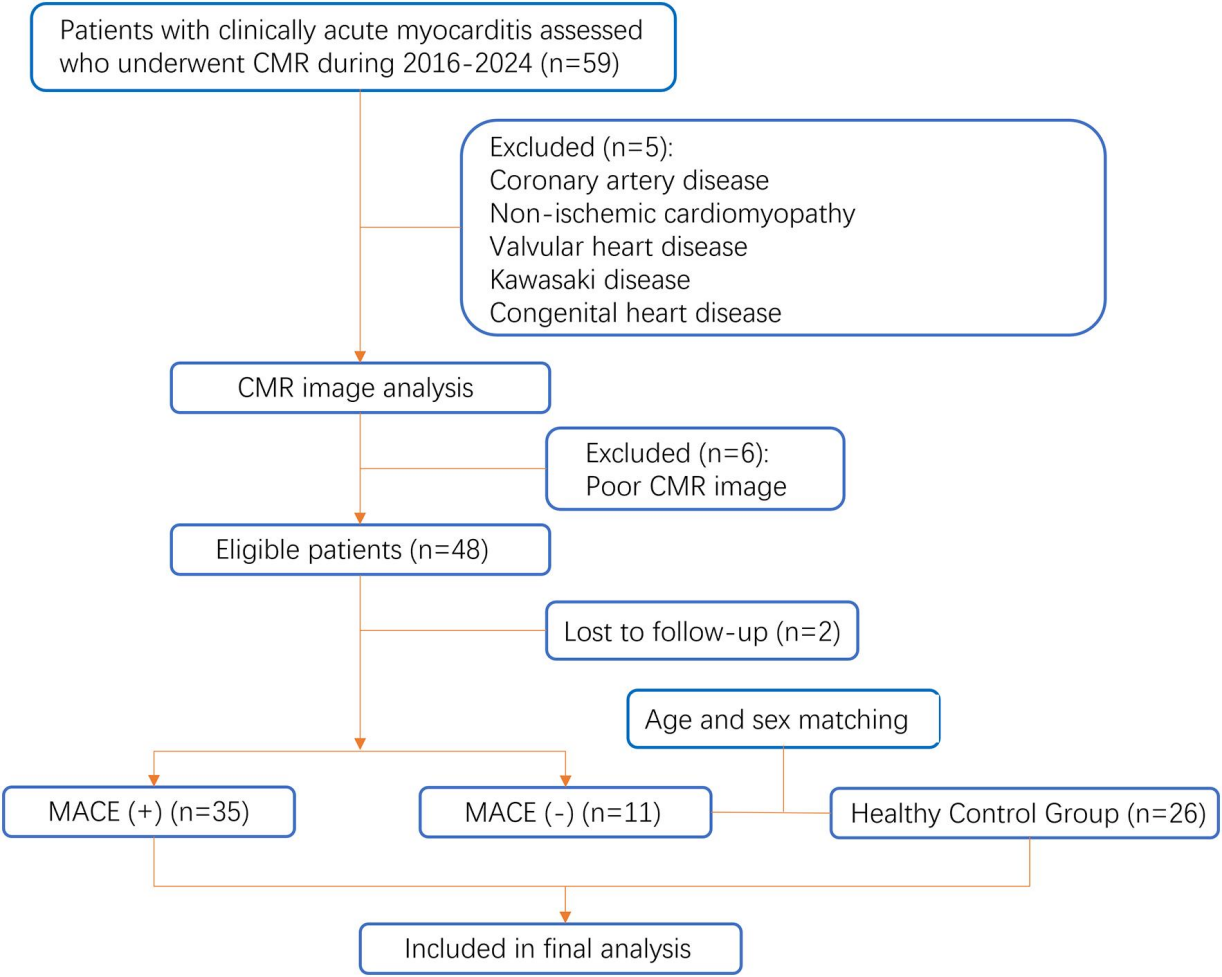
Flow chart of the study population.

Twenty-six healthy volunteers who were age and gender matched, had no history of cardiovascular disease, normal ECG results, and no abnormal manifestations on CMR examination were selected as the healthy control group. The flow chart of the study is shown in Figure 1.

### 2.2 CMR protocols

CMR acquisition was performed using a SIEMENS MAGNETOM 1.5 T scanner (SIEMENS Healthcare, USA) equipped with an 8-channel phased array cardiac coil. The patient was in the supine position during acquisition. The CMR scanning protocol included cine imaging and late gadolinium enhancement (LGE) imaging. True Fast Imaging with Steady-state Precession (TrueFISP) end-expiratory breath-hold cine images were acquired to obtain 25 long-axis two- and four-chamber views and 9–11 short-axis slices covering the entire left ventricle from the mitral annulus to the apical epicardium. Typical cine imaging parameters were as follows: repetition time (TR)/echo time (TE), 48 ms/1.5 ms; flip angle (FA), 55°; matrix, 170 × 208; field of view (FOV), 256 × 209 mm; and slice thickness, 8 mm. 10–15 min after intravenous bolus injection of 0.2 mmol/kg Gd-DTPA (Bayer Magnevist, Berlin, Germany), myocardial LGE images were acquired using a Phase Sensitive Inversion Recovery (PSIR). Long-axis and short-axis view images were acquired at the same locations as the cine images. The parameters of the LGE sequence were as follows: TR/TE/FA, 804 ms/1.4 ms/45°; matrix, 143 × 256; FOV, 350 × 273 mm; slice thickness, 8 mm.

### 2.3 CMR image post processing

Image post-processing was performed by a radiologist (RC.R.) with 2 years of experience in interpreting CMR images using commercial Cvi42 software (version 6.1.2, Circle, Calgary, Canada). A cohort of 19 patients was randomly selected to assess interobserver agreement (with another radiologist (WT.L.) with 2 years of CMR experience) prior to feature tracking analysis. The first radiologist also repeated the analysis after a 2-week interval to assess intraobserver agreement. Each radiologist (the two radiologists mentioned, RC.R., WT.L.) reviewed all LGE images for the presence or absence of nonischemic myocardial injury. When there was any difference opinion, agreement was reached after discussion with among three another radiologists (RC.R., WT.L. and Y.Z.) with 20 years of experience).

### 2.4 LA, LV volume and function analysis

End-systole and end-diastole of the LV in short-axis cine images were automatically defined, as well as the endocardium and epicardium of the LV in each section from the apical to the mitral valve plane. Papillary muscles were included in the LV volume. The LV volume and functional parameters were calculated and normalized to body surface area (BSA): LV end-diastolic volume index (LVEDVI), LV end-systolic volume index (LVESVI), LV stroke volume index (LVSVI), and LV mass index (LVMI).

The end-diastolic phase of all cine images (including short-axis and 2-chamber, 3-chamber, and 4-chamber views) was selected for overall LV strain analysis. The software automatically calculated LV global radial strain (LVGRS), LV global circumferential strain (LVGCS), and LV global longitudinal strain (LVGLS).

**Figure 2:**
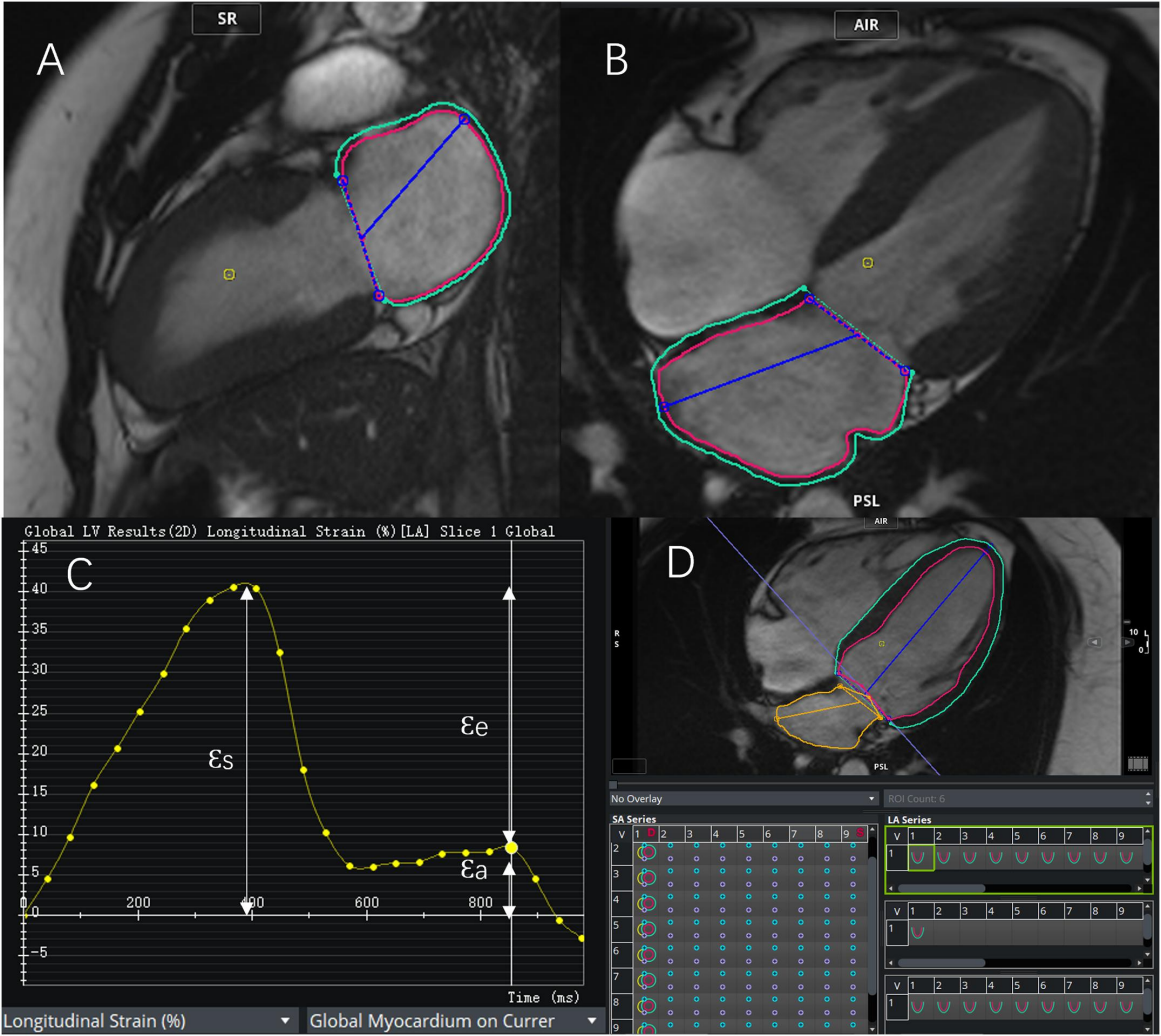
Schematic of left atrial and left ventricular strain and volume analysis. **(A,B)** Left atrial (LA) volume and strain were assessed based on two-chamber **(A)** and four-chamber **(B)** cine images using the biplane equation. **(C)** Representative LA longitudinal strain curve illustrating reservoir strain (εs), conduit strain (εe), and booster strain (εa).**(D)** Left ventricular (LV) volume and strain parameters were derived from short-axis and long-axis cine images.

LA volume and function analysis were performed using cine images in 2-chamber and 4-chamber views. The inner edge of the LA (excluding LA appendages and pulmonary veins) was defined in three phases: LV end-systole (LAVmax) and end-diastole before (LAVpre) and after (LAVmin) LA contraction. These were normalized based on BSA to obtain the LAVmax index and the LAVpre index. LA functional parameters, including total LAEF, passive LAEF, and active LAEF. Also in the 2-chamber and 4-chamber views of the cine images, the left atrial border was manually depicted at the end of LV systole. LV strain metrics, including reservoir strain (εs), conduit strain (εe), and booster strain (εa), were automatically derived from the longitudinal strain curves. The LACI was calculated as the ratio of LAVmin to LVEDV and expressed as a percentage. The above analysis is shown in Figure 2.

### 2.5 MAPSE measurement

**Figure 3:**
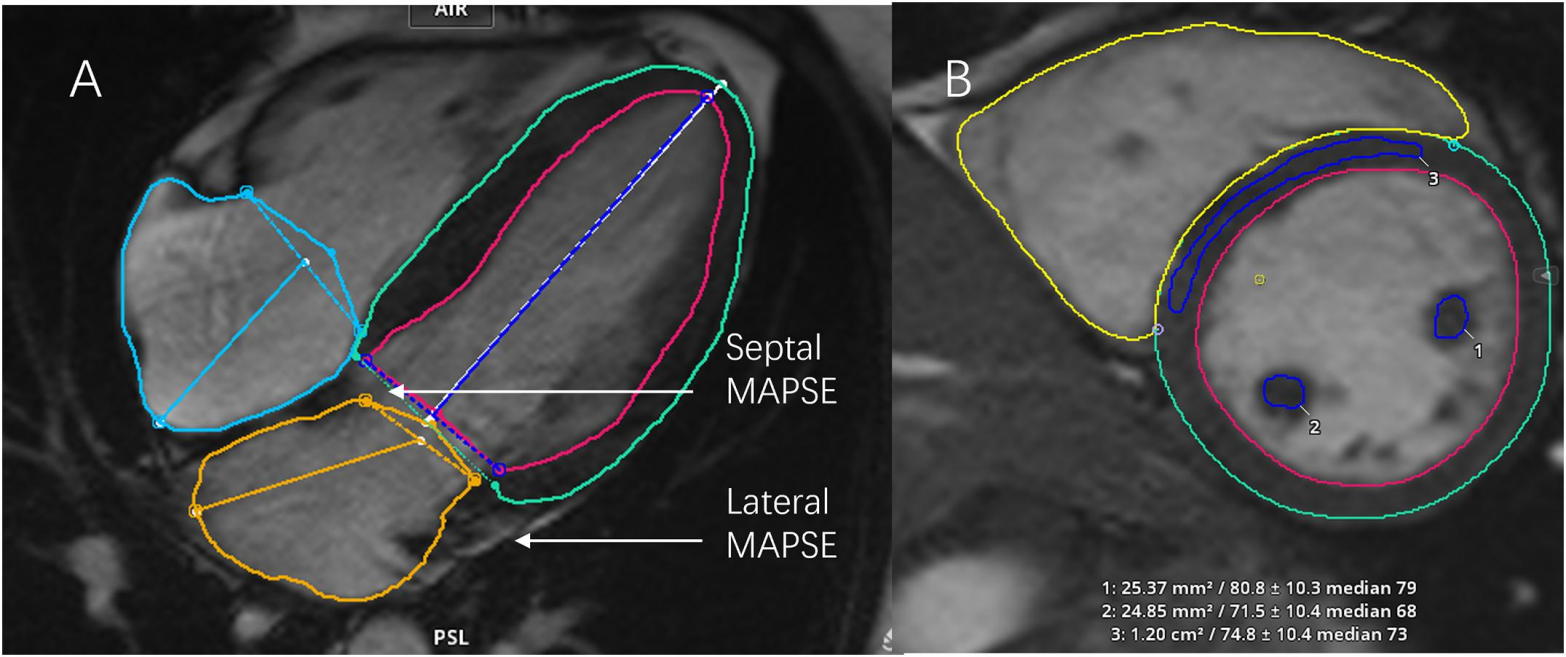
Measurement of mitral annular plane systolic excursion (MAPSE) and papillary muscle signal intensity. **(A)** Illustration of MAPSE measurement on a four-chamber cine image. The mitral annular plane was tracked to measure the displacement of the lateral (Lateral MAPSE) and septal (Septal MAPSE) mitral annulus from end-diastole to end-systole. **(B)** Analysis of papillary muscle signal intensity on a short-axis cine image. Regions of interest were drawn on the anterior and posterior papillary muscles and the interventricular septum to calculate the anterior papillary signal ratio (APS) and posterior papillary signal ratio (PPS).

The end-diastolic and end-systolic mitral annular planes were defined in cine images of the 4-chamber view, MAPSE was automatically measured. The mitral annular tracking points were adjusted on an image-by-image basis. Lateral MAPSE was defined as the distance from end-systole to end-diastole at the lateral attachment point in the mitral valve. Septal MAPSE was defined as the distance from end-systole to end-diastole at the attachment point of the mitral valve in the septum. The above analysis is shown in Figure 3.

### 2.6 Signal ratio of the papillary muscle measurement

The papillary muscle signals were measured at end-systole by selecting the short-axis cine view. The regions of interest of the left ventricular anterior papillary muscle, posterior papillary muscle, and interventricular septum were manually plotted separately. The inclusion of the left ventricular cavity signal was avoided as much as possible. Finally, the anterior papillary muscle signal, posterior papillary muscle signal, and left ventricular septum signal were compared to obtain the anterior papillary signal ratio (APS) and posterior papillary signal ratio (PPS), respectively. The above analysis is shown in Figure 3.

### 2.7 Statistical analysis

All data were analysed using SPSS Statistics (Version-26.0; IBM Corp) and GraphPad Prism (Version-10.2; GraphPad Software Inc.). Continuous variables are expressed as mean ± standard deviation, and for normal distributions, comparisons were made using independent samples *t*-tests. For non-normal distributions, expressed as the median and interquartile range (IQR), comparisons were made using the Mann–Whitney *U* test. Categorical variables were expressed as counts and percentages and were compared using chi-square or Fisher's exact tests as needed. The occurrence of adverse clinical endpoints in the high- and low-risk groups was assessed using Kaplan–Meier survival analyses after setting MACE high/low risk cut-offs using ROC curve analysis. Graphs were compared using the log-rank test. Associations of CMR parameters with MACE were determined using univariate and multivariate Cox proportional risk regression analyses. The proportional hazards assumption was verified using Schoenfeld residuals, and the assumption was met for all included variables. Parameters with *P* values <0.05 in univariate analyses were included in multivariate analyses. Results are reported as risk ratios (HR) and 95% confidence intervals (CI). Correlations between LV strain, LA strain, LACI, and MAPSE were determined using linear regression. Reproducibility analyses were performed using intragroup correlation coefficients (ICC) to determine inter- and intra-observer agreement. *P*-values <0.05 were considered statistically significant.

## 3 Results

### 3.1 Study population

This study retrospectively analyzed 46 patients with acute myocarditis and 26 age- and sex-matched healthy people as controls. After a median follow-up period of 740 (IQR: 513.4–966.6) days, 11 patients (23.91%) with MACE, including cardiac death (3 patients, 6.52%), cardiogenic shock (4 patients, 8.70%), and rehospitalization due to recurrence of myocarditis or exacerbation of heart failure (4 patients, 8.70%).

### 3.2 Clinical characteristics and baseline data of patients with and without MACE and normal controls

Baseline characteristics are summarized in Table 1. Heart rate was higher in patients with MACE, with no significant difference between the two comparisons. In addition, hs-TNI levels were higher in patients without MACE (0.92 ng/mL [IQR: 0.04–3.39 ng/mL] vs. 0.02 ng/mL [IQR: 0.01–0.96 ng/mL]).

**Table 1:** Baseline characteristics.

Characteristics	All Patients (*N* = 46)		Control group (*N* = 26)	*P* value
	MACE(−) (*N* = 35)	MACE(+) (*N* = 11)		
Demographic characteristics				
Age, years	28 (17, 39)	32 (20, 61)	26 (18, 40)	0.600
Sex, male, *N* (%)	25 (71.4)	6 (54.5)	15 (57.7)	0.430
Body mass index, kg/m^2^	23.54 ± 4.56	24.07 ± 5.11	24.88 ± 3.83	0.502
Heart rate, beat/min	66 (58, 102)	80 (70, 91)	74 (69, 81)	0.015
Cardiovascular risk factors				
Hypertension, *N* (%)	4 (11.4)	3 (27.3)	–	0.333
Diabetes mellitus, *N* (%)	2 (5.7)	2 (18.2)	–	0.238
Laboratory results				
NT-proBNP, pg/mL	1,151 (141, 1,747)	826 (173, 3,363)	–	0.780
hs-TNI, ng/mL	0.92 (0.04, 3.39)	0.02 (0.01, 0.96)	–	0.018
WBC count, × 10^9^/L	8.47 (6.92, 12.03)	9.07 (6.3, 13.66)	–	0.975
CK, U/L	97 (51, 161)	93 (65, 191)	–	0.844

MACE, major adverse cardiac events; NT-proBNP, N-terminal pro-B-type natriuretic peptide; hs-TNI, high-sensitivity cardiac troponin I; WBC, white blood cells; CK, creatine kinase. For variables available in all three groups (Age, Sex, BMI, Heart rate), *P*-values represent the comparison among the three groups (MACE+, MACE-, and Control) using One-way ANOVA, Kruskal–Wallis test, or Chi-square test. For variables unavailable in the Control group (Hypertension, Diabetes mellitus, and laboratory results), *P*-values represent the comparison between MACE(+) and MACE(–) groups using Mann–Whitney *U* test or Fisher's exact test.

### 3.3 Cardiac magnetic resonance-derived LA, LV volume and functional parameters and mitral annular displacement and papillary muscle signal intensity analysis

CMR parameters are shown in Table 2. LVSVI and LVEF were significantly lower in patients with MACE than in normal controls (29.07 ± 11.34 mL/m^2^ vs. 38.64 ± 8.32 mL/m^2^ and 37.47% [IQR: 20.74–56.98%] vs. 57.10% [IQR: 54.17–60.18%], respectively). LV strain parameters (LVGLS, LVGCS, LVGRS) were significantly lower in patients with MACE than in normal controls vs. patients who did not develop MACE (−9.16 ± 3.03% vs. −16.8 ± 3.55% vs. −15.85 ± 6.69%, respectively, and −8.7% [IQR: −14.3- −6.6%] vs. −19.0% [IQR. −20.4–16.5%] vs. −17.8% [IQR: −19.2–12.8%], 13.8% [IQR: 8.3–21.7%] vs. 32.2% [IQR: 25.8–36.0%] vs. 29.9% [IQR: 19.1–33.7%]). In contrast, LV strain parameters in patients without MACE were not significantly different from normal controls.

**Table 2:** CMR parameters.

Parameters	All Patients (*N* = 46)		Control group (*N* = 26)	*P* value
	MACE(-) (*N* = 35)	MACE(+) (*N* = 11)		
LV parameters				
LVEDVI, mL/m^2^	63.11 (55.65, 87.6)	72.63 (65.11, 108.00)	69.84 (58.3, 79.31)	0.534
LVESVI, mL/m^2^	28.43 (24.64, 41.84)	52.77 (29.41, 67.52)	30.35 (23.79, 34.32)	0.156
LVSVI, mL/m^2^	33.48 ± 10.61	29.07 ± 11.34	38.64 ± 8.32^b^	0.022
LVEF, %	53.19 (46.93, 59.39)	37.47 (20.74, 56.98)	57.10 (54.17, 60.18)^b^	0.008
LVMI, g/m^2^	44.40 (40.39, 58.40)	43.24 (39.97, 56.14)	43.63 (35.88, 50.60)	0.321
LVGLS, %	−15.85 ± 6.69	−9.16 ± 3.03^a^	−16.8 ± 3.55^b^	0.001
LVGCS,%	−17.8 (−19.2, −12.8)	−8.7 (−14.3, −6.6)^a^	−19.0 (−20.4, −16.5)^b^	0.001
LVGRS, %	29.9 (19.1, 33.7)	13.8 (8.3, 21.7)^a^	32.2 (25.8, 36.0)^b^	0.002
Presence of LGE	19 (0.54)	5 (0.45)	–	0.734
LA parameters				
LAVImax, mL/m^2^	30.44 ± 9.99	28.72 ± 11.57	29.28 ± 8.09	0.830
LAVImin, mL/m^2^	12.24 (8.21, 16.75)	9.20 (7.75, 21.8)	9.64 (6.74, 12.53)	0.160
LAVIpre, mL/m^2^	19.75 (13.88, 25.82)	17.23 (12.12, 26.55)	17.17 (12.75, 23.34)	0.500
Total LAEF, %	57.40 ± 12.99	52.52 ± 15.53	66.64 ± 7.18^a^^,^^b^	0.001
Passive LAEF, %	34.24 ± 9.48	28.39 ± 14.54	39.75 ± 7.41^b^	0.005
Booster LAEF, %	35.97 ± 12.96	33.84 ± 16.03	44.62 ± 10.18^a^	0.014
εs, %	28.3 (16.7, 36.3)	18.2 (15.2, 20.6)^a^	34.5 (28.8, 47.8)^a^^,^^b^	0.001
εe, %	15.3 (10.3, 24.1)	8.6 (5.8, 12.0)^a^	23.0 (18.6, 32.3)^a^^,^^b^	0.001
εa, %	12.1 (8.1, 13.6)	7.8 (6.2, 9.8)	11.8 (9.7, 13.6)	0.112
LACI, %	18.18 ± 7.27	16.64 ± 6.40	14.95 ± 5.87	0.181
MAPSE				
Septal MAPSE, mm	14.51 ± 4.04	9.24 ± 3.20^a^	17.33 ± 3.06^a^^,^^b^	0.001
Lateral MAPSE, mm	9.97 (8.88, 11.01)	6.98 (3.91, 8.55)^a^	13.53 (11.44, 14.9)^a^^,^^b^	0.001
Papillary muscle signal ratio				
APS	1.05 (0.90, 1.14)	1.04 (0.97, 1.10)	1.06 (0.99, 1.12)	0.615
PPS	1.06 (0.91, 1.19)	1.12 (0.96, 1.21)	1.04 (0.93, 1.17)	0.673

LV, left ventricular; LVEDVI, left ventricular end diastolic volume index; LVESVI, left ventricular end systolic volume index; LVSVI, left ventricular stroke volume index; LVEF, left ventricular ejection fraction; LVMI, LV mass index; LVGLS, left ventricular global longitudinal strain; LVGCS, left ventricular global circumferential strain; LVGRS, left ventricular global radial strain; LA, left atrial; LAVImax, maximum left atrial volume index; LAVImin, minimum left atrial volume index; LAVIpre, left atrial volume index at end-diastole before LA contraction; LAEF, left atrium ejection fraction; εs, reservoir strain; εe, conduit strain; εa, booster strain; LACI, left atrioventricular coupling index; MAPSE, mitral annular plane systolic excursion; APS, anterior papillary signal ratio; PPS, posterior papillary signal ratio.

^a^ Compared with MACE(+).

^b^ Compared with MACE(+).

For LA volume and functional parameters, total LAEF was significantly lower in patients with MACE (57.40 ± 12.99% vs. 52.52 ± 15.53% vs. 66.64 ± 7.18%) and passive LAEF was significantly lower in patients with MACE (34.24 ± 9.48% vs. 39.75 ± 7.41%), whereas booster LAEF was significantly lower in patients without MACE (35.97 ± 12.96% vs. 44.62 ± 10.18%).

For LA strain parameters, εs and εe were significantly lower in patients without MACE and in patients with MACE compared with normal controls (28.3% [IQR: 16.7–36.3%] vs. 18.2% [IQR: 15.2- 20.6%] vs. 34.5% [IQR: 28.8 −47.8%], 15.3% [IQR: 10.3- 24.1%] vs. 8.6% [IQR: 5.8–12.0%] vs. 23.0% [IQR: 18.6–32.3%]). For both groups of patients with acute myocarditis, εs and εe were significantly impaired in patients.

For mitral annular displacement, septal MAPSE and lateral MAPSE were significantly lower in patients with MACE compared with the other two groups (14.51 ± 4.04 mm vs. 9.24 ± 3.20 mm vs. 17.33 ± 3.06 mm, 9.97 mm [IQR: 8.88–11.01 mm] vs. 6.98 mm [IQR. 3.91–8.55 mm] vs. 13.53 mm [IQR: 11.44–14.9 mm]).

There was no significant difference in the signal ratio of the papillary muscles between the three groups.

### 3.4 Diagnostic performance of myocardial strain parameters and MAPSE for MACE in patients with myocarditis

**Figure 4:**
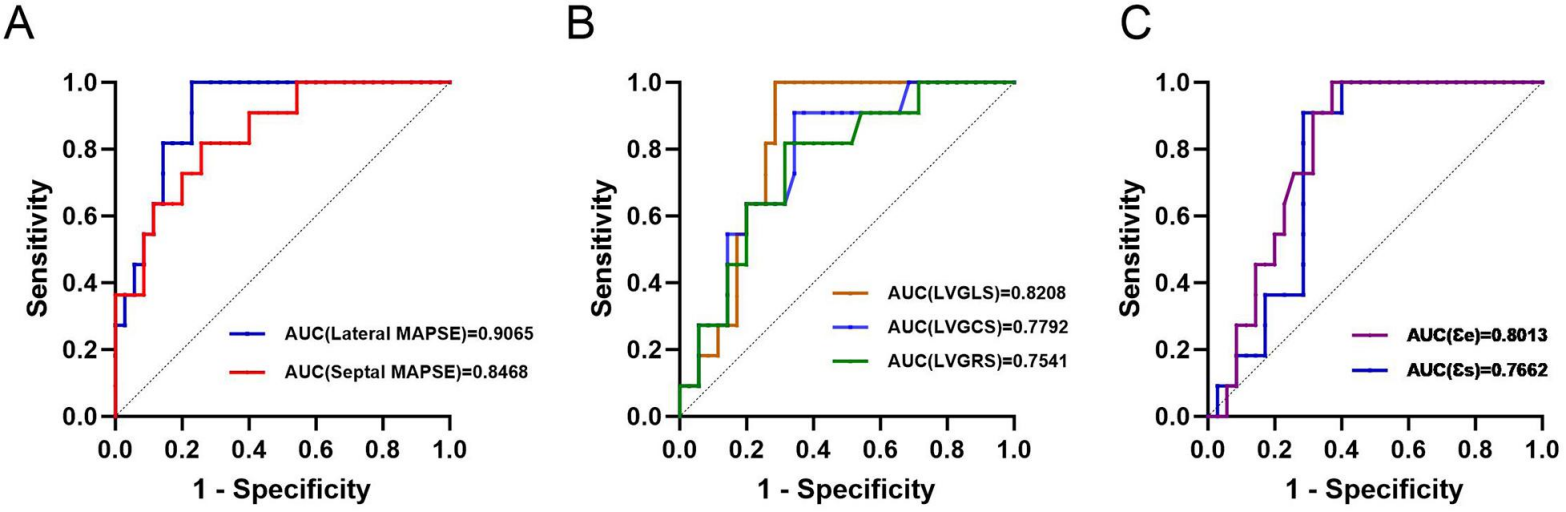
Diagnostic performance of MAPSE **(A)**, left ventricular strain **(B)**, and left atrial strain **(C)** for adverse cardiovascular events in patients with myocarditis.

We evaluated the diagnostic performance of myocardial strain parameters and MAPSE in identifying adverse cardiovascular events in patients with myocarditis using ROC curve analysis. Results showed (Figure 4) good diagnostic performance of left ventricular strain, left atrial reservoir and conduit strain, and MAPSE. The AUC was higher for septal MAPSE and lateral MAPSE.

**Figure 5:**
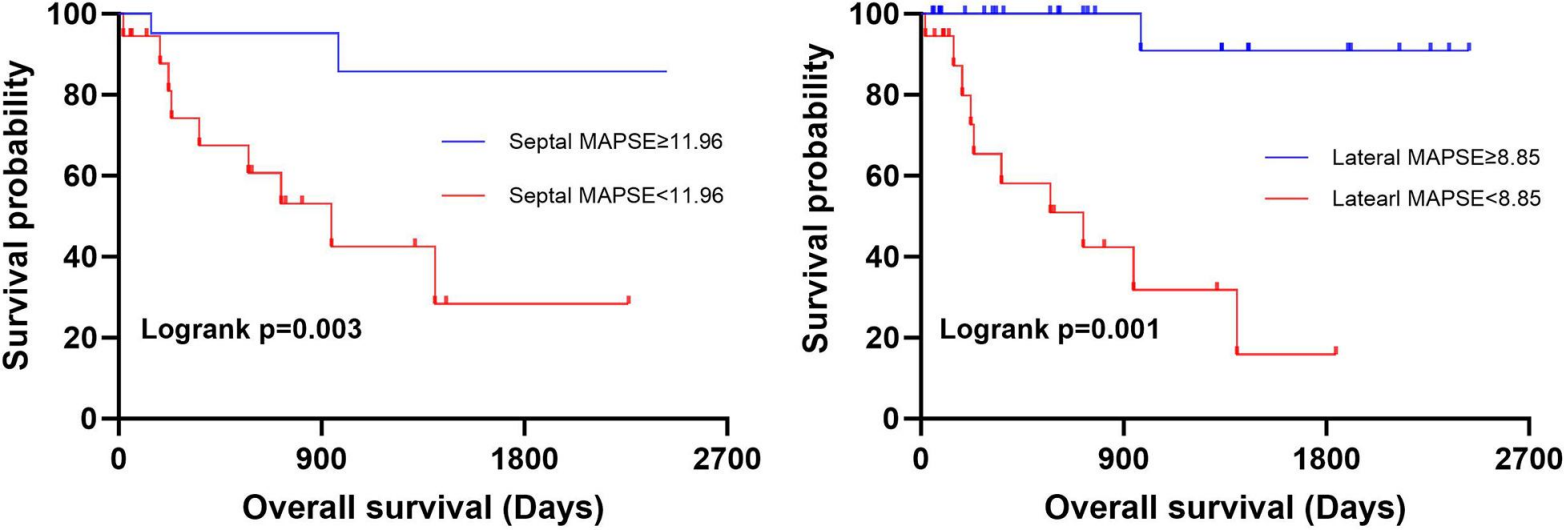
Prognostic value of MAPSE. Optimal lateral MAPSE **(A)** and optimal septal MAPSE **(B)** cutoffs of 11.96 mm and 8.85 mm (log-rank test *P* = 0.0025, *P* = 0.0065).

We determined the optimal lateral MAPSE of 11.96 mm and the optimal septal MAPSE cutoff value (Youden index) of 8.85 mm, respectively, in the entire cohort to better categorize the patients into low-risk and high-risk groups (Figure 5).

Table 3 shows the results of univariate and multivariate Cox regression analyses. In univariate analysis, age, εe, septal MAPSE, and lateral MAPSE were significantly associated with MACE occurrence (HR 1.044, 95% CI 1.006–1.084; HR 0.872, 95% CI 0.761–1.000; HR 0.755, 95% CI 0.627–0.909; HR 0.707, 95% CI 0.568–0.880). These were selected for multifactorial analysis, which showed that age, septal MAPSE and lateral MAPSE were independent predictors of MACE (HR 1.141, 95% CI 1.058–1.231; HR 0.673, 95% CI 0.453–0.998; HR 0.544, 95% CI 0.333–0.890; respectively).

**Table 3:** Univariate and multivariate Cox regression analyses used to predict MACE.

Variables	Univariable analysis		Multivariable analysis	
	Hazard ratio (95% CI)	*P* value	Hazard ratio (95% CI)	*P* value
Age, years	1.044 (1.006, 1.084)	0.022	1.139 (1.056, 1.228)	0.001
Heart rate, beat/min	1.025 (0.996, 1.055)	0.092		
LVEDVI, mL/m^2^	1.005 (0.983, 1.027)	0.683		
LVESVI, mL/m^2^	1.008 (0.991, 1.026)	0.358		
LVSVI, mL/m^2^	0.959 (0.904, 1.018)	0.172		
LVEF, %	0.981 (0.947, 1.015)	0.267		
LVGLS, %	1.140 (1.015, 1.281)	0.027	0.867 (0.660, 1.139)	0.306
LVGCS,%	1.123 (0.999, 1.261)	0.051		
LVGRS, %	0.947 (0.893, 1.003)	0.064		
Presence of LGE	1.722 (0.523, 5.681)	0.373		
LAVImax, mL/m^2^	0.973 (0.911, 1.039)	0.410		
LAVImin, mL/m^2^	1.004 (0.940, 1.072)	0.900		
LAVIpre, mL/m^2^	1.000 (0.935, 1.069)	0.995		
Total LAEF, %	0.986 (0.951, 1.021)	0.426		
Passive LAEF, %	0.955 (0.903, 1.008)	0.097		
Booster LAEF, %	0.995 (0.955, 1.036)	0.797		
εs, %	0.931 (0.859, 1.008)	0.079		
εe, %	0.872 (0.761, 1.000)	0.050	0.898 (0.706, 1.142)	0.381
εa, %	0.928 (0.799, 1.078)	0.327		
LACI, %	0.995 (0.913, 1.085)	0.909		
Septal MAPSE, mm	0.755 (0.627, 0.909)	0.003	0.647 (0.420, 0.995)	0.047
Lateral MAPSE, mm	0.707 (0.568, 0.880)	0.002	0.594 (0.355, 0.995)	0.048
APS	3.341 (0.079, 141.023)	0.528		
PPS	6.980 (0.256, 190.324)	0.249		

LVEDVI, left ventricular end diastolic volume index; LVESVI, left ventricular end systolic volume index; LVSVI, left ventricular stroke volume index; LVEF, left ventricular ejection fraction; LVGLS, left ventricular global longitudinal strain; LVGCS, left ventricular global circumferential strain; LVGRS, left ventricular global radial strain; LAVImax, maximum left atrial volume index; LAVImin, minimum left atrial volume index; LAVIpre, left atrial volume index at end-diastole before LA contraction; LAEF, left atrium ejection fraction; εs, reservoir strain; εe, conduit strain; εa, booster strain; LACI, left atrioventricular coupling index; MAPSE, mitral annular plane systolic excursion; APS, anterior papillary signal ratio; PPS, posterior papillary signal ratio.

**Figure 6:**
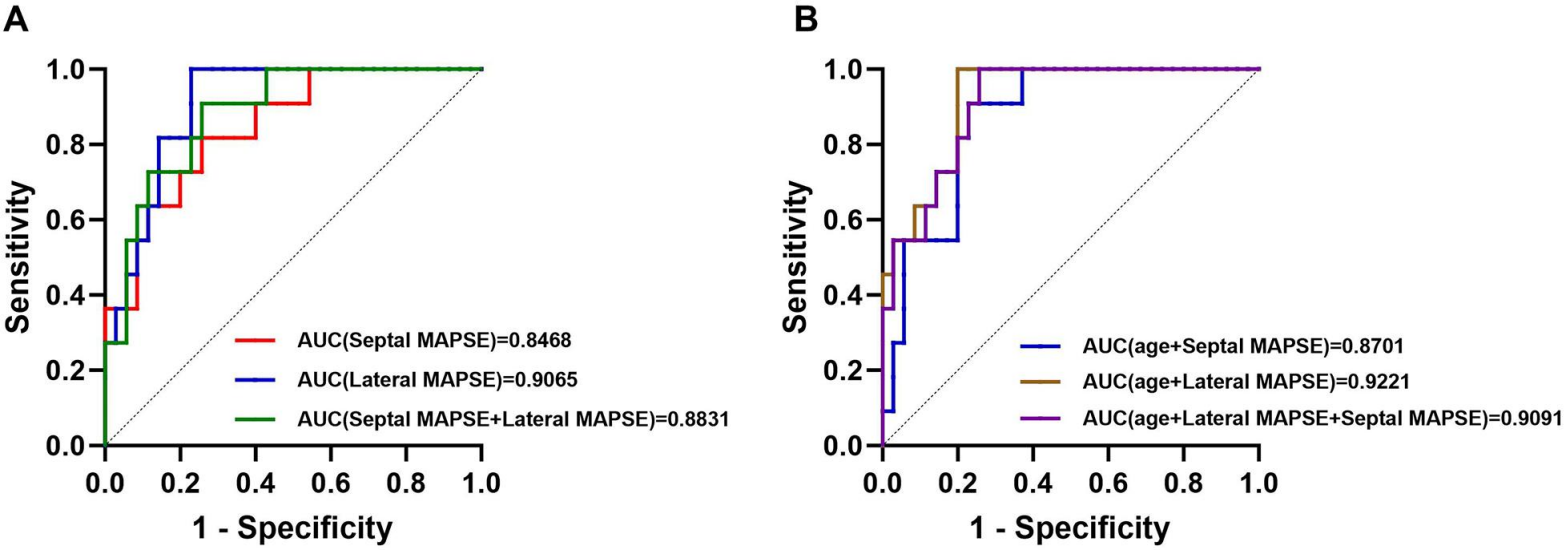
Predictive value of different Cox regression models for adverse cardiovascular events in patients with myocarditis. **(A)** Receiver operating characteristic (ROC) curves comparing the diagnostic performance of Septal MAPSE, Lateral MAPSE, and the combination of both. Lateral MAPSE alone yielded the highest Area Under the Curve (AUC = 0.9065) compared to Septal MAPSE (AUC = 0.8468) and the combined model (AUC = 0.8831). **(B)** ROC curves comparing multivariate models incorporating Age. The model combining Age and Lateral MAPSE demonstrated the highest predictive value (AUC = 0.9221), which was superior to Age + Septal MAPSE (AUC = 0.8701) and slightly higher than the model combining Age + Lateral MAPSE + Septal MAPSE (AUC = 0.9091).

We combined variables with independent predictive value from multifactor Cox regression analysis into different Cox regression models. The results (Figure 6) showed that the model with both lateral and septal MAPSE did not improve predictive performance compared with lateral MAPSE alone (AUC = 0.8831 vs. AUC = 0.9095). Figure 6 showed that the model with the addition of age had a higher predictive value (AUC = 0.8701 vs. AUC = 0.8468, AUC = 0.9221 vs. AUC = 0.9065, and AUC = 0.9091 vs. AUC = 0.8831), but similarly, the addition of interventricular MAPSE did not improve predictive performance with the addition of age plus lateral MAPSE compared to interventricular MAPSE (AUC = 0.9091 vs. AUC = 0.9221).

### 3.5 Correlation between MAPSE and other CMR parameters in patients and control group

MAPSE was moderately correlated with LA and LV strain parameters. Septal MAPSE and LACI were poorly correlated. Lateral MAPSE did not correlate significantly with LACI. MAPSE did not correlate significantly with any of the papillary muscle signal ratios. The details of correlations were presented in Table 4.

**Table 4:** Correlation between MRI parameters and MAPSE.

Parameters	Septal MAPSE		Lateral MAPSE	
	*r*	*P*	*r*	*P*
LVEF, %	0.335	0.004	0.493	0.001
LVGLS, %	−0.644	0.001	−0.595	0.001
LVGCS, %	−0.560	0.001	−0.537	0.001
LVGRS, %	0.519	0.001	0.482	0.001
LGE, n	0.051	0.737	−0.157	0.296
εs, %	0.552	0.001	0.615	0.001
εe, %	0.547	0.001	0.597	0.001
εa, %	0.378	0.001	0.427	0.001
LACI, %	−0.253	0.032	−0.137	0.252
APS	0.108	0.368	0.030	0.801
PPS	0.029	0.809	−0.108	0.369

LVEF, left ventricular ejection fraction; LVGLS, left ventricular global longitudinal strain; LVGCS, left ventricular global circumferential strain; LVGRS, left ventricular global radial strain; εs, reservoir strain; εe, conduit strain; εa, booster strain; LACI, left atrioventricular coupling index; MAPSE, mitral annular plane systolic excursion; APS, anterior papillary signal ratio; PPS, posterior papillary signal ratio.

### 3.6 Reproducibility analysis

As shown in Table 5, LA and LV strains and MAPSE showed excellent intraobserver and interobserver agreement.

**Table 5:** Intra- and inter-observer variability of MRI parameters.

Parameters	Intraobserver		Interobserver	
	ICC (95% CI)	*P* Value	ICC (95% CI)	*P* Value
LVGLS, %	0.958 (0.896, 0.984)	<0.001	0.928 (0.826, 0.972)	<0.001
LVGCS, %	0.981 (0.953, 0.993)	<0.001	0.959 (0.797, 0.987)	<0.001
LVGRS, %	0.992 (0.980, 0.997)	<0.001	0.989 (0.972, 0.996)	<0.001
εs, %	0.975 (0.937, 0.990)	<0.001	0.984 (0.959, 0.994)	<0.001
εe, %	0.972 (0.922, 0.990)	<0.001	0.976 (0.940, 0.991)	<0.001
εa, %	0.830 (0.618, 0.931)	<0.001	0.913 (0.792, 0.965)	<0.001
Septal MAPSE, mm	0.981 (0.953, 0.993)	<0.001	0.985 (0.962, 0.994)	<0.001
Lateral MAPSE, mm	0.987 (0.966, 0.995)	<0.001	0.984 (0.960, 0.994)	<0.001

LVGLS, left ventricular global longitudinal strain; LVGCS, left ventricular global circumferential strain; LVGRS, left ventricular global radial strain; εs, reservoir strain; εe, conduit strain; εa, booster strain; MAPSE, mitral annular plane systolic excursion.

## 4 Discussion

This study focused on the prognostic value of mitral annular plane systolic excursion in acute myocarditis and compared it with left atrial and left ventricular strain. The main findings were as follows: (1) Compared with normal controls, septal MAPSE and lateral MAPSE were decreased in patients with acute myocarditis. Compared with patients without MACE, septal MAPSE and lateral MAPSE were impaired in patients with MACE. (2) Compared with LA and LV strains, septal MAPSE and lateral MAPSE had higher diagnostic performance. (3) Septal MAPSE and lateral MAPSE can be used as prognostic factors for major adverse cardiovascular events in patients with acute myocarditis, and the predictive value of lateral MAPSE is even higher. (4) Septal MAPSE and lateral MAPSE have a good correlation with LA and LV strain.

The ventricular myocardium consists of an obliquely traveling endocardium and epicardium and a circumferentially traveling intermediate myocardium (15). One of the main purposes of the shear deformation of the left ventricle during contraction is to amplify the 15% shortening of the cardiomyocytes into a 40% radial left ventricular wall thickening, which in turn leads to a change in the left ventricular ejection fraction of >60% in a normal heart (16). Thus, the long-axis function of the heart plays an important role in the ejection activity of the heart. This may also explain the excellent diagnostic performance of global longitudinal strain (GLS) and global circumferential strain (GCS) in myocarditis (17), as well as in patients with acute myocarditis with preserved ejection fraction (18, 19). Throughout the cardiac cycle, the mitral annulus is pulled toward the apex, and the left ventricular cavity volume decreases, which prompts ventricular ejection. At this time the base of the atrium moves downward and the volume of the atrium increases thereby drawing blood from the pulmonary veins into the atria. During atrial contraction, the mitral annulus is pulled off the apex by the contracting atrial myocardium, further contributing to atrial emptying and ventricular filling (20–22). Basically, MAPSE is the result of contraction of both subendocardial and subepicardial longitudinal fibers (23). It has been demonstrated that up to 60% of the per-passage output can be explained by the movement of the longitudinal mitral annulus (24, 25).LVGLS has a strong correlation and MAPSE is significantly impaired in patients with acute myocarditis. In our study, although LVGLS was significantly associated with MACE in the univariate analysis, it lost its significance in the multivariate model, whereas MAPSE remained an independent predictor. Both parameters evaluate longitudinal myocardial function and are inherently collinear. The superiority of MAPSE in our multivariate model may be attributed to its methodological robustness. LVGLS, derived from CMR feature tracking, heavily relies on optimal image quality and accurate endocardial border definition, which can be compromised by severe myocardial edema and late gadolinium enhancement typical of acute myocarditis. Conversely, MAPSE is a simple, direct anatomical measurement of annular displacement that is highly reproducible and less susceptible to image artifacts, making it a more stable prognostic marker in this specific cohort.

Measurement of MAPSE in patients with acute or chronic myocardial infarction using different echocardiographic methods has shown that MAPSE predicts the occurrence of adverse events (26, 27). Ultrasound is more sensitive to noise, which can be affected by the different angles of manipulation and inexperience of the operator (28).In contrast, CMR is more accurate and reproducible and does not require additional sequences and specific Software. The displacement of the mitral annulus relative to the apex measured by CMR was found to correlate well with the overall longitudinal strain measured by echocardiography (29).The present study demonstrated good intraobserver and interobserver agreement for CMR-derived MAPSE.

The present study demonstrated that both lateral and septal MAPSE have independent predictive value for adverse events in acute myocarditis, with lateral MAPSE having a higher value and a greater risk ratio. Of note, the predictive value of lateral and septal MAPSE was diminished when both were included in the prediction model compared with lateral MAPSE alone. Rangarajan et al. demonstrated that lateral MAPSE, as a surrogate for LV long-axis function, was an independent predictor of major adverse cardiovascular events using a CMR routine movie sequence (14). Although LVEF was lower in the MACE group, MAPSE represents longitudinal function, which is governed by subendocardial fibers that are often more susceptible to ischemia and inflammation than the circumferential fibers responsible for LVEF. Therefore, MAPSE may serve as a more sensitive, early marker of myocardial dysfunction even before significant drops in ejection fraction occur. A large multicenter study demonstrated that lateral MAPSE is a significant independent predictor of mortality in patients with LV dysfunction and reduced ejection fraction and can provide more valuable prognostic information than conventional imaging indices, including ejection fraction and late gadolinium enhancement of the LGE (13). Differently, Mayr et al. found that septal MAPSE, compared with lateral and mean MAPSE, had a higher ST-segment-elevation MACE predictive value in patients with ST-segment-elevation myocardial infarction predictive value of MACE (30).This may be because lateral MAPSE is mainly influenced by left ventricular long-axis function, whereas septal MAPSE is also influenced by right ventricular reciprocal function. There are fewer data on the prevalence and prognostic significance of right ventricular involvement in patients with acute myocarditis. Aquaro et al. recruited 27 (17.8%) of 151 patients with clinically suspected acute myocarditis with signs of right ventricular involvement, and multivariate Cox regression analysis showed that right ventricular involvement was an independent predictor of cardiac events (31). Bernhard et al. included 659 patients with suspected myocarditis, and 162 (25.5%) had right ventricular involvement., with 162 (25.9%) patients showing impaired RVEF and 144 (21.9%) patients with impaired RV GLS, and multivariate Cox regression showed that right ventricular GLS provided only limited value in suspected myocarditis (32).Based on the results of the present study, we recommend the use of lateral MAPSE independent of right ventricular involvement to predict the occurrence of adverse cardiovascular events in patients with acute myocarditis.

Both left ventricular and left atrial strains were significantly reduced in patients who developed MACE in this study compared with patients with acute myocarditis who did not develop MACE, and the ROC curves showed good discriminatory performance for adverse events for both of these strain parameters, whereas multifactorial cox regression did not show an independent predictive value, which we speculate may be related to the small sample size.

It has been found that the papillary muscle to LV wall myocardial signal ratio is low in patients with mitral valve prolapse, which has not been observed in other conditions that may affect the mitral annulus (33). This “dark papillary muscle sign” is an independent predictor of poor prognosis in patients with ventricular arrhythmias and normal LVEF, and may be due to a transient perfusion defect of the muscle during peak ventricular contraction (34). We analyzed the papillary muscle signal ratio, and our results showed that there was no significant correlation between the papillary muscle signal ratio and MAPSE. The reason may be that myocarditis is a disease that primarily involves the myocardium, and the papillary muscles are rarely involved. High signal in the myocardium is less frequently observed on cine sequences, and even less frequently in the septum. Deux et al. performed scanning cine, enhancement cine, and delayed gadolinium enhancement scans in 18 patients with acute myocarditis and showed that areas of high signal were detected on the scanning cine sequences in 28% (5/18) of the patients (35). Therefore, a larger cohort should be conducted in the future to explore the value of papillary muscle signal ratio in the diagnosis and prognosis of myocarditis.

This study has several limitations. First, this was a retrospective study spanning from 2016 to 2024. Advanced parametric mapping sequences (T1 and T2 mapping) were not routinely available or performed for all patients, particularly in the earlier years. Thus, the diagnosis relied on the 2013 ESC criteria and standard Lake Louise Criteria rather than the updated 2018 criteria. However, LGE and cine imaging were available for all patients to ensure robust functional and structural analysis. Second, the sample size and the number of MACE events (*n* = 11) in this single-center study were limited. Although this raises the risk of overfitting in the multivariate Cox regression, acute myocarditis cohorts with long-term CMR follow-up are inherently small. To mitigate this, we strictly limited the number of covariates, an approach consistent with similar recent studies (e.g., Lee et al. (10)). Nonetheless, these multivariate results should be considered exploratory and require validation in larger, multi-center cohorts. Third, the prolonged enrollment period (2016–2024) introduces potential confounding effects, as the evolution of heart failure pharmacotherapy over this 8-year span may have influenced individual clinical outcomes.

## 5 Conclusion

CMR-derived mitral annular systolic displacement has prognostic value for adverse cardiovascular events in patients with acute myocarditis, and correlates well with the left atrium and left ventricle. It can be used as a surrogate index of left ventricular function to evaluate the prognosis of patients with acute myocarditis.

## Data Availability

The raw data supporting the conclusions of this article will be made available by the authors, without undue reservation.
